# Intra-Arterial Lidocaine Blunts the Trigeminocardiac Reflex during Endovascular Treatment of a Carotid-Cavernous Fistula

**DOI:** 10.1155/2021/2342347

**Published:** 2021-01-05

**Authors:** Renee L. Coleman, Dmitri Bezinover, Douglas C. Jones, Kevin M. Cockroft, Uma R. Parekh

**Affiliations:** ^1^Penn State Milton S. Hershey Medical Center, Department of Anesthesiology & Perioperative Medicine, Hershey, PA, USA; ^2^Penn State Milton S. Hershey Medical Center, Department of Neurosurgery, Hershey, PA, USA

## Abstract

Carotid-cavernous fistulas (CCFs) are vascular shunts that allow blood to flow from the carotid artery or its branches into the cavernous sinus. Endovascular embolization is the treatment modality of choice. The trigeminocardiac reflex (TCR) is a vagally mediated reflex that can lead to hemodynamic instability. It can be activated during embolization procedures due to the proximity of vagal efferent neurovascular structures within the cavernous sinus. This case report describes the intraoperative management of recurrent, profound bradycardia due to TCR during endovascular CCF embolization.

## 1. Introduction

The trigeminocardiac reflex (TCR) is a well-described brainstem reflex known to complicate neurosurgical, maxillofacial, and oral surgery [1]. It elicits from stimulation of any sensory branch of the trigeminal nerve and can manifest as either a tachyarrhythmia or bradycardia, with asystole, an extreme presentation [2, 3]. The overall incidence of TCR is 11% but is higher in the case of trigeminal nerve stimulation from the arterial side [1]. Intravenous atropine administration has been recommended to blunt TCR [1]. In this case report, we present a novel strategy to prevent the cardiovascular effects associated with TCR activation in a patient undergoing endovascular embolization of a cavernous carotid fistula (CCF). Written authorization was obtained from the patient for submission of this case report for potential publication.

## 2. Case Description

A 61-year-old Caucasian male with past medical history significant for well-controlled hypothyroidism and obesity (BMI 34) presented for endovascular embolization of a right-sided CCF. Diagnostic angiogram demonstrated connections from dural branches of the right internal carotid artery and middle meningeal branch of right external carotid artery (ECA) to an indirect fistula of the right cavernous sinus. Venous drainage was enabled predominantly through the right superior ophthalmic vein.

Preoperatively, the patient was hemodynamically stable (blood pressure and heart rate were 139/90 mmHg and 67 beats/min, respectively) with an ECG demonstrating normal sinus rhythm. Physical examination revealed an abducens nerve palsy. Standard American Society of Anesthesiologists monitors were applied in the operating room and femoral arterial access was established by the surgeon. Anesthesia induction was uneventful using 50 *μ*g of fentanyl, 180 mg of propofol, and 50 mg of rocuronium to facilitate intubation performed with video laryngoscopy. To optimize neuromonitoring conditions, anesthesia was maintained using a combination of inhaled sevoflurane, infusions of propofol (80–150 *μ*g/kg/min), and dexmedetomidine (0.03–0.05 *μ*g/kg/h), as well as intermittent fentanyl administration.

Vascular access for embolization was attempted by the neurosurgical team, first via the right femoral vein, planning for a transvenous approach through the right inferior petrosal sinus. This attempt was, however, unsuccessful. An attempt through the right superior ophthalmic vein also failed. The interventional team finally opted for a transarterial approach from the femoral artery to the right middle meningeal artery branch of the ECA. Once the site of the fistula was reached, an angiogram was performed using 110 mL of Omnipaque. This resulted in profound bradycardia that progressed to asystole. The injection was stopped and normal sinus rhythm returned spontaneously after approximately 40 seconds. We hypothesized that the profound bradycardia was triggered by activation of the TCR with the Omnipaque injection. Before the next injection, the patient was pretreated with glycopyrrolate 0.2 mg IV, and the depth of anesthesia increased by increasing the inspired sevoflurane concentration. During the second attempt, the patient again became asystolic, which resolved spontaneously after 10–15 seconds. It was felt that direct stimulation of either the dura or the trigeminal nerve in the cavernous sinus was responsible for activation of the TCR with subsequent asystole. After discussing management options, it was decided to prophylactically inject 0.5 mL of lidocaine 1% directly into the middle meningeal branch of the ECA through the indwelling catheter before the next angiography sequence. This successfully prevented the development of bradycardia during angiography. The angiogram was completed which demonstrated complete fistula occlusion. It was postulated that the combination of the contrast itself (being viscous and thrombogenic), along with the brief period of hypotension during asystole, resulted in thrombosis of the vessels feeding the fistula. The patient was extubated neurologically intact and transported to the neurocritical care unit for recovery and postoperative monitoring. On postoperative day (POD) 1, his only complaints were of a minor headache and right-sided perioral numbness. The patient was discharged home on POD 2 with improvement of his symptoms, all of which resolved a few days later. Repeat diagnostic angiogram performed four months after procedure demonstrated no residual CCF.

## 3. Discussion

TCR is manifested as a sudden onset of dysrhythmias, hypotension, and apnea. TCR has been described during intracranial, maxillofacial, ophthalmic surgery, microcompression, endovascular treatment of intracranial dural arteriovenous, and carotid-cavernous fistulas as well as radiofrequency lesioning of the trigeminal ganglion [1]. It can also be associated with hypermotility of the gastrointestinal tract [2].

TCR can also occur during treatment of abnormal vascular shunts such as CCFs. CCFs allow blood to flow either directly or indirectly from the carotid artery, or its branches, into the cavernous sinus. Endovascular embolization is considered to be the treatment of choice due to its good outcomes and minimal risk profile [4].

**Figure 1:**
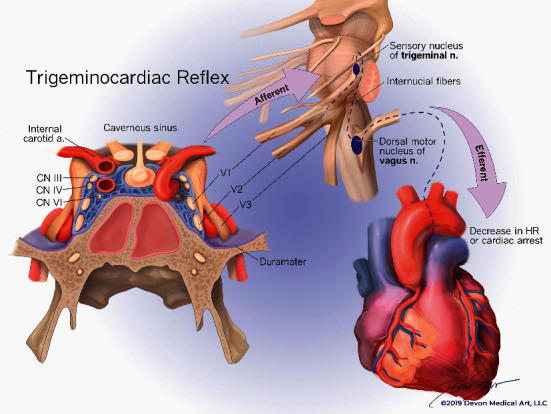
The trigeminocardiac reflex arc via branches of the trigeminal nerve in the cavernous sinus. TCR could also be initiated by stimulation of the dura mater supplied by branches of the trigeminal nerve.

The risks associated with TCR activation should always be considered when an intervention is performed in the territory of the trigeminal nerve. First and second divisions of the trigeminal nerve and other important neural and vascular structures, including the internal carotid artery, oculomotor nerve, trochlear nerve, and abducens nerve, traverse the cavernous sinus (Figure 1). TCR is associated with activation of sensory fibers of the trigeminal nerve with subsequent transmission via the Gasserian ganglion to the sensory nucleolus. After a number of intermediate connections, a signal transmits to the dorsal motor nucleus of the vagal nerve with simultaneous activation of cardioinhibitory parasympathetic and sympathetic vagal neurons which results in bradycardia, vasodilatation, and, frequently, hemodynamic instability [2, 5]. It has been demonstrated that TCR can cause severe bradycardia and asystole during endovascular embolization of a CCF, especially during ethylene vinyl alcohol (Onyx) and dimethyl sulfoxide (DMSO) injection [6, 7]. Onyx is a nonadhesive liquid embolic agent made up of an ethylene vinyl alcohol copolymer dissolved in DMSO with suspended micronized tantalum powder to provide contrast for visualization under imaging [1, 8]. Direct application of DMSO on the trigeminal nerve can result in mechanical irritation and neurotoxicity [9]. In our case, TCR occurred after the Omnipaque injection, before any injection with Onyx/DMSO. Omnipaque (Iohexol) is a second-generation nonionic, water-soluble, radiocontrast agent used in radiological imaging procedures. Reported adverse effects include headache, nausea, vomiting, seizures, meningeal and radicular irritation, myoclonic spasms, rhabdomyolysis, prolonged reversible paraplegia after intrathecal injection, and acute anaphylaxis after intra-arterial injection. Direct toxic effects have not been described [10]. It has been previously demonstrated that the TCR can be associated with thermal and electrical stimuli, mechanical stretch, higher resting parasympathetic tone, light plane of anesthesia, hypercapnia, and hypoxemia [5, 11] as well as with the use of several medications, including opioids (alfentanil and sufentanil), beta blockers, and calcium channel blockers [11, 12]. Mechanical stimulation of the dura mater, innervated by the branches of the trigeminal nerve or direct stimulation of the trigeminal nerve divisions in the cavernous sinus, was likely responsible for the initiation of TCR in our case.

Meuwly et al. concluded that hemodynamic instability is more pronounced when TCR is triggered during high-risk procedures under a light plane of anesthesia. The authors recommend careful monitoring of anesthesia depth during high-risk surgery [11]. Other factors predisposing activation of TCR include hypoxia, hypercapnia, and acidosis. If the reflex occurs during a surgical procedure, cessation of manipulation, administration of anticholinergic drugs (atropine or glycopyrrolate), and epinephrine, as well as increasing the depth of anesthesia using intravenous and/or volatile anesthetics have been recommended [5].  Table 1 summarizes the preventive and therapeutic measures.

In our case, administration of glycopyrrolate and deepening the anesthetic with sevoflurane failed to abolish the TCR. Considering that the intra-arterial catheter was in close proximity of the ophthalmic and the maxillary divisions of the trigeminal nerve in the cavernous sinus, we administered lidocaine through this catheter. Previous studies have demonstrated the efficacy of topical local anesthetic application and local anesthetic nerve blocks in preventing TCR [11]. Topical lidocaine has been shown to suppress the reflex during open craniotomy for microvascular decompression of the trigeminal nerve [13]. The occulocardiac reflex, a type of trigeminocardiac reflex, has been shown to be blunted with peribulbar block with bupivacaine in patients undergoing ophthalmic surgery [14]. Campbell et al. have suggested that local anesthetic infiltration and nerve blocks during maxillofacial surgery can prevent TCR [15].

Lidocaine is a local anesthetic that can be used locally or regionally to produce a temporary loss of sensory, motor, and autonomic function. It is also used as an antiarrhythmic agent. Lidocaine binds to sodium channels within neurocellular membranes. This temporarily blocks sodium influx into the cell preventing membrane depolarization [16]. Although all cellular membranes are affected, sensory nerve fibers are usually affected first because they are much thinner and can be easily penetrated [16, 17]. Lidocaine also blocks potassium and calcium channels, as well as G-protein-coupled acetylcholine, glutamate, and opioid receptors [16]. Lidocaine has an excellent safety profile when limited to a dose of less than 4.5 mg/kg depending on the site of injection and rate of absorption. In our case, injection of only 5 mg through the arterial catheter in the cavernous sinus completely prevented TCR without any adverse effects. To our knowledge, this is the first case reporting the efficacy of intra-arterial lidocaine in blunting TCR during endovascular neurosurgery.

In conclusion, after several failed attempts to prevent TCR, injection of 5 mg of intra-arterial lidocaine through an indwelling catheter was successful in preventing a recurrence of TCR and was associated with no adverse effects. When the potentially devastating complications associated with TCR are compared to the good safety profile of intra-arterial lidocaine administration, this approach can be recommended for use during surgical procedures where there is a risk of TCR activation.

## Figures and Tables

**Table 1:** Prevention and treatment of trigeminocardiac reflex.

Awareness of occurrence during high-risk surgery
Increasing the depth of anesthesia with intravenous or volatile agents before surgical stimulation
Prophylactic nerve blocks were appropriate
Gentle manipulation and traction around the nerve
Avoidance of hypoxia and hypercapnia
Close monitoring of heart rate and blood pressure
Cessation of stimulation if the reflex is triggered
Treatment with vagolytic agents (atropine or glycopyrrolate)
If unresponsive to vagolytic agents, epinephrine is administered
Prophylaxis or treatment with intravascular lidocaine through an indwelling catheter during endovascular procedures.

## Data Availability

All data supporting the conclusion can be accessed online. Patient data cannot be accessed due to HIPPA requirements.

## References

[1] Lv X., Li Y., Jiang C., Wu Z. (2010). The incidence of trigeminocardiac reflex in endovascular treatment of dural arteriovenous fistula with onyx. *Interventional Neuroradiology*.

[2] Schaller B. (2004). Trigeminocardiac reflex. A clinical phenomenon or a new physiological entity?. *Journal of Neurology*.

[3] Fowler S. J., Featherston M. (2004). Recurrent atrial tachyarrhythmia triggered by percutaneous balloon rhizotomy of the trigeminal nerve. *Anaesthesia and Intensive Care*.

[4] Zanaty M., Chalouhi N., Tjoumakaris S. I., Hasan D., Rosenwasser R. H., Jabbour P. (2014). Endovascular treatment of carotid-cavernous fistulas. *Neurosurgery Clinics of North America*.

[5] Chowdhury T., Mendelowith D., Golanov E. (2015). Trigemino-cardiac reflex examination G: Trigeminocardiac reflex: the current clinical and physiological knowledge. *Journal of Neurosurgical Anesthesiology*.

[6] Wang J., Wu H. C., Wang W. W. (2016). Trigeminal cardiac reflex caused by onyx embolization of intracranial dural arteriovenous fistula. *Turkish Neurosurgery*.

[7] Puri A. S., Thiex R., Zarzour H., Rahbar R., Orbach D. B. (2011). Trigeminocardiac reflex in a child during pre-Onyx DMSO injection for juvenile nasopharyngeal angiofibroma embolization. A case report. *Interventional Neuroradiology*.

[8] Amiridze N., Darwish R. (2009). Hemodynamic instability during treatment of intracranial dural arteriovenous fistula and carotid cavernous fistula with Onyx: preliminary results and anesthesia considerations. *Journal of NeuroInterventional Surgery*.

[9] Larsen J., Gasser K., Hahin R. (1996). An analysis of dimethylsulfoxide-induced action potential block: a comparative study of DMSO and other aliphatic water soluble solutes. *Toxicology and Applied Pharmacology*.

[10] Canbay O., Bal N., Akinci S., Kanbak M., Aypar U. (2004). Rhabdomyolysis after intraoperative myelography. *Pediatric Anesthesia*.

[11] Meuwly C., Chowdhury T., Sandu N., Reck M., Erne P., Schaller B. (2015). Anesthetic influence on occurrence and treatment of the trigemino-cardiac reflex: a systematic literature review. *Medicine*.

[12] Chung C. J., Lee J. M., Choi S. R., Lee S. C., Lee J. H. (2008). Effect of remifentanil on oculocardiac reflex in paediatric strabismus surgery. *Acta Anaesthesiologica Scandinavica*.

[13] Chigurupati K., Vemuri N. N., Velivela S. R., Mastan S. S., Thotakura A. K. (2013). Topical lidocaine to suppress trigemino-cardiac reflex. *British Journal of Anaesthesia*.

[14] Shende D., Sadhasivam S., Madan R. (2000). Effects of peribulbar bupivacaine as an adjunct to general anaesthesia on peri-operative outcome following retinal detachment surgery. *Anaesthesia*.

[15] Campbell R., Rodrigo D., Cheung L. (1994). Asystole and bradycardia during maxillofacial surgery. *Anesthesia Progress*.

[16] Hermanns H., Hollmann M. W., Stevens M. F. (2019). Molecular mechanisms of action of systemic lidocaine in acute and chronic pain: a narrative review. *British Journal of Anaesthesia*.

[17] Vadhanan P., Tripaty D., Adinarayanan S. (2015). Physiological and pharmacologic aspects of peripheral nerve blocks. *Journal of Anaesthesiology Clinical Pharmacology*.

